# The effects of post activation potentiation warm-up and pre-shot routine programs on driving performance in amateur golfers

**DOI:** 10.1371/journal.pone.0240881

**Published:** 2020-10-20

**Authors:** Wichai Yeemin, Supatcharin Kemarat, Apiluk Theanthong

**Affiliations:** Faculty of Allied Health Sciences, Thammasat University, Bangkok, Thailand; Tongii University, CHINA

## Abstract

The purpose of this study was to assess the effects of three different programs, i.e. active dynamic warm-up program plus functional resistance warm-up using Theraband plus pre-shot routine program (AFPR); pre-shot routine program (PR); and active dynamic warm-up program plus functional resistance warm-up using Theraband (AF) on driver club head speed, driving distance, and driving accuracy in the amateur golfers. Fifteen amateur golfers with an average age of 19.67 ± 0.89 years and 4.87 ± 1.77 points of average handicap were assigned to participate in either AFPR, PR or AF program. All participants in the three programs practiced three sessions on non-consecutive days per week during the intervention phase. Each participant’s performance was assessed before and after six weeks of the program through hitting ten maximal drives with the ball flight and swing analyzed using the P3ProSwing Golf Simulator and recorded for the driver club head speed, driving distance, and driving accuracy. Multivariate analysis of variance showed no statistically significant differences (P < .05) of the performances of the golfers participated in the 3 programs (club head speed: F = 1.02, P = 0.33; accuracy: F = 0.32, P = 0.72; distance: F = 0.18, P = 0.83). Furthermore, a paired t-tests also showed no statistically significant (P < .05) improvement occurred in the 3 programs after the six-week training. Although the effect of the 3 programs did not show statistically significant increase in the performance of the amateur golfers, however, the three parameters of the performance, i.e. the driver club head speed, the driving distance and the driving accuracy showed certain improvements. The 3 training programs may have benefit to the amateur golfers with certain increases of their performance.

## Introduction

Golf is an extremely popular sport played across the world regardless of gender, skill level or age. It also involves a wide variety of shots to master (e.g., driving, chipping, and putting) [1]. Extended periods between shots, and competitive situations that could be distracting and destructive performance [2]. Therefore, golf presents participants with both cognitive and behavioral challenges [3]. Successful golfers have been identified as having the ability to hit the ball effectively. An effective driving depends on many factors such as driver club head speed, driving distance, and driving accuracy. These factors can be developed through the thorough physical and mental training programs. For example, flexibility, power, and strength training can improve driving distance and driver club head speed [4]. A short-term activation in a warm-up program can improve driving distance and driver club head speed [5]. Moreover, sports science studies found that warming-up combining with post-activation potentiation (PAP) technique can improve the efficiency of the movement [6–10]. The effect of utilizing the PAP technique combined with a warm-up on golf performance can be seen by Tilley and Macfarlane’s study [11]. The study found that Active Dynamic warm-up program PLUS 10-minute functional resistance warm-up using Theraband leads to a significant increase in immediate performance of certain factors of the golf drive such as driving distance, drivers club head speed, and driving accuracy. In addition to utilizing the PAP technique combined with a warm-up, effective driving can be improved by controlling the psychological state while hitting the ball. However, it was usually found that the golfers had the difficulty to control the psychological state during the competition due to the stress and the anxiety that they had to face. Tanaka and Sekiya [12] revealed that the pressure condition increased physiological arousal, decreased the amplitudes of arms and club movements on the backswing, and decreased the club movement speed on the fore-swing. One specific cognitive-behavioral strategy used in golfing is a pre-performance routine or pre-shot routine [13]. The use of pre-shot routines is effective in improving the performance of skilled participants across several sports [2, 13–24]. Particularly, the pre-shot routine that focuses on both physical and mental (imagery, breathing control, and self-talk) preparation will be a good effect on driving performance [13].

As mentioned above, it could be assumed that the development of an effective driving could be achieved through the collaboration among the active dynamic warm-up, the post-activation potentiation (PAP) technique, and the pre-shot routine that focused on physical and mental preparation. Currently, however, there has been no studies on the effect of utilizing dynamic warm-up programs combined with the post-activation potentiation (PAP) technique and the pre-shot routine on driving performance of the golfers. Therefore, the purpose of this study was to assess the effects of three different programs, i.e. active dynamic warm-up program plus functional resistance warm-up using Theraband plus pre-shot routine program (AFPR), pre-shot routine program (PR), and active dynamic warm-up program plus functional resistance warm-up using Theraband (AF) on driver club head speed, driving distance and driving accuracy in the amateur golfers. It was hoped that the results of the study can be used as data and guidelines for improving and increasing the efficiency of driving of the golf players.

## Materials and methods

### Participants

The Ethics Committee of Thammasat University certified that the study was accomplished according to the Declaration of Helsinki as revised in 2013. The G*Power software version 3.1 was used to determine the sample size by using data of Tilley and Macfarlane’s study [11]. The following parameters were selected: large effect size (f = 0.85), an alpha level of .05, a power level of 0.8, and three groups. The total sample size was determined to be at least 12. However, the total sample size of this study was 15 in case of any dropout of the participants and to conform to the Tilley and Macfarlane’s study [11]. Participants in this study included 15 amateur golfers (14 males, 1 female) who were an average of 19.67 ± 0.89 years of age and 4.87 ± 1.77 of the golf club handicap (Table 1). All participants met the inclusion criteria which included being a member of the Thammasat University golf club, aged between 18–23 years old, possessing an official golf club handicap at the time of the study between 0–10, competing for at least 3 times per year in the golf events organized by the public or private sector, no current physical injuries, no medical problem history of the upper and the lower limbs during 3 months before the start of the study, be willing to participate in the study, no barriers to communication. All participants were assigned either to AFPR (n = 5) or PR (n = 5) or AF (n = 5) group by sorting the average value of club head speed, which was derived from 10 times driving (thirty-second rest intervals between each shot) and randomly assigned to the experimental groups.

**Table 1:** Participant characteristics.

Experimental group	N	Age (yrs.)	Handicap	Experience (yrs.)
AFPR	5	20.20±1.10	4.80±1.64	8.20±1.64
PR	5	19.60±0.55	5.00±2.55	7.00±0.71
AF	5	19.20±1.10	4.80±1.48	8.40±1.52
Total	15	19.67±0.89	4.87±1.77	7.87±1.46

### Measurements

The pre- and post-test of golf driving measurements were analyzed by a P3ProSwing Golf Simulator located in a golf lab at the Sports Science and Sports Development Department, Thammasat University. The participant performed a full swing and hit a real golf ball to a screen. The screen presented a fairway, on which the ball was placed. It also presented a green and a hole with a pin and a flag. A visual trajectory line of the golf ball flied to the final position was displayed on the screen.

The golf ball was shot from a 22.9 cm x 35.6 cm sensing platform with 1.5 cm high artificial grass on top. The platform contained 65 optical sensors that detected the information about the speed and direction of the club head at the ball impact. The simulator estimated the distance and direction for each shot. The simulator accurately monitored the ball flight with 99% precision [25].

### Procedures

For each golf shot, the distance, the club head speed at the ball impact [11] and the accuracy in terms of the direction [25] were measured. The collection of data of the pre-test was one week before participating in the program and the post-test was one week after participating in the program. All participants used their own drivers. They were informed that the same driver and under the same conditions would apply for the post-test. Before the pre-test measurement started, they could take practice shots with their drivers to familiarize themselves with the new surface and the artificial environment. At the start of the measurement, the participants were instructed to aim for the pin and to proceed at their own pace. All golfers performed 10 test shots with thirty-second rest intervals between each shot. The same procedure was used during the post-test. After the pre-test, the participants attended the study venue on three separate, non-consecutive days (maximum of 1 hour required at each session) over a maximum of 6-week period at the Sports Science and Sports Development Department, Thammasat University. The set up for the exercises was identical at each session for each participant. At every session, there was at least one researcher present for safety and supervising the program with the participants. The participants carried out one of the three programs shown below (Table 2), during each session:

**Table 2:** Experimental programs.

Activity details	Experimental group		
	AFPR	PR	AF
**Active dynamic warm-up program PLUS functional resistance warm-up using Theraband**	✓	**-**	✓
1. Running on a treadmill at 60% of maximum heart rate for 5 minutes			
2. Theraband (red) exercises (Each exercise conducted for 10 times and repeated for 2 sets with 30-second rest between set).			
• trunk rotation movement in standing • standing lunge and trunk rotation movement • right arm cross chest adduction and internal rotation with body rotation • left arm external rotation and shoulder abduction with rotation • wood chops from the right and left trunk rotation			
3. Ten practice swing shots with a driver (slow to speed pace and 30-second rest between shot).			
**Pre-shot routine program**	✓	✓	**-**
1. Taking a deep breath with tempo 1-2-3-4 during inhalation and 8-7-6-5-4-3-2-1 during exhalation and aiming to the pin			
2. Performing the practice swings at their own pace			
3. Addressing the ball and the set-up position, grip checking, and eye contact with the ball.			
4. Aiming to the pin and taking a deep breath with tempo 1-2-3-4 during inhalation and 8-7-6-5-4-3-2-1 during exhalation			
5. Performing the full swing (back swing, down swing, follow through)			
**Twenty practice swings shot with a driver in the Golf Simulator**	✓	✓	✓

### Statistical analysis

A Shapiro-Wilk test was conducted to verify if all the data met the normality test assumption. Comparing driver club head speed, driving distance and driving accuracy among the three programs by using a multivariate analysis of variance MANOVA test [26, 27]. Paired t-tests were then performed between each of the three sets of data to identify within-group differences between the data for each program [25]. An alpha value of p < 0.05 was set as the criterion level of significance.

## Results

The subjects in this study were tested for driver club head speed, driving distance, and driving accuracy after AFPR, PR and AF treatments. The result of MANOVA revealed no statistically significant differences for the driver club head speed (p = 0.33), driving distance (p = 0.83), and driving accuracy (p = 0.72) between the three programs (Table 3). Paired t-tests between pre and post-treatment were used to further investigate the three factors of performance that showed no statistically significant differences. The result showed that no statistically significant differences of the three factors were seen between the three programs for the pre- and post-tests. However, the mean results did show improved figures for these three performance factors with post-intervention. The analysis showed that the AFPR had increased driving distance by 4.1 yards (p = 0.70) and driving accuracy by 3.87 yards over the pre-treatment (p = 0.82). The PR had increased the driver club head speed by 0.19 miles/hour (p = 0.82), the driving distance by 10.79 yards (p = 0.17), and the driving accuracy by 1.18 yards compared to the pre-treatment (p = 0.90). The AF had increased the driver club head speed by 0.32 miles/hour (p = 0.66), the driving distance by 22.39 yards (p = 0.05), and the driving accuracy by 22.60 yards (p = 0.11) over the pre-treatment (Table 4).

**Table 3:** Comparison of treatments on the golf performance variables after 6 weeks of training.

	Experimental group			F	P
	AFPR	PR	AF		
Club head speed	110.47±5.33	107.78±6.00	103.19±10.22	1.02	0.33
Distance	259.89±16.18	257.21±16.48	251.93±28.11	0.18	0.83
Accuracy	23.58±20.58	30.85±22.33	21.29±14.66	0.32	0.72

**Table 4:** Comparison of the golf performance variables prior to training and after 6 weeks of training.

**Club head speed**				
**Experiment group**	**Prior to training**	**After 6 weeks**	**t**	**P**
AFPR	110.63±5.53	110.47±5.33	0.33	0.75
PR	107.59±7.01	107.78±6.00	-0.23	0.82
AF	102.87±9.75	103.19±10.22	0.47	0.66
**Distance**				
**Experiment group**	**Prior to training**	**After 6 weeks**	**t**	**P**
AFPR	255.79±12.53	259.89±16.18	-0.40	0.70
PR	246.42±27.52	257.21±16.48	-1.64	0.17
AF	229.54±34.27	251.93±28.11	-2.77	0.05
**Accuracy**				
**Experiment group**	**Prior to training**	**After 6 weeks**	**t**	**P**
AFPR	27.45±26.09	23.58±20.58	0.23	0.82
PR	32.03±9.80	30.85±22.33	0.13	0.90
AF	43.89±27.45	21.29±14.66	2.04	0.11

## Discussion

The purpose of this study was to assess the effects of three different programs, i.e. AFPR, PR and AF on driver club head speed, driving distance and driving accuracy in amateur golfers. The results of this study revealed improvement in the driver club head speed, the driving distance, and the driving accuracy when compared to the pre-intervention. As there has been no previous research on the performance of utilizing dynamic warm-up programs combined with the post-activation potentiation (PAP) technique and the pre-shot routine on later driving performance, therefore, it is difficult to draw a comparison to the other studies. Driving performance has been shown to have a positive relationship with the driver club head speed, the driving distance, and the driving accuracy. Several studies have used these factors as an indicator of driving performance [4, 11, 28, 29]. The development of each factor can be achieved through physical and mental training. For the club head speed, it can be achieved through training warm-up programs [5–8, 11, 28] and pre-physical and mental routine [13, 14, 16]. However, there is no study focuses on a combination of both programs. Therefore, this study tried to explore the effect of training warm-up and pre-shot routine together and compared to solely train either warm-up program or pre-shot routine program. The result of this study showed that the three programs did not significantly affect the club head speed. The difference between this study and the previous research is the duration of the test in which conducted after ending the one-week programs. This may be a reason why training the three programs in this study led to little improvement in the parameters of the driving performance. Usually, the study of the effect of pre-shot routine and warm-up programs were done in an acute phase, which was tested immediately after the end of the program [6, 7, 11, 13]. An increase in the training period for more than six weeks could be a technique to obtain a different result. Cohn et al. suggested that the training routine of physical and mental skills should be practiced for 16 weeks or more because it would show the change in the performance of the golf players [2]. Although there was no significant difference after the sixth weeks, however, this study found that the club head speed was increased in the PR and AF groups. This suggested that the three programs developed as part of this study helped to increase the club head speed score. This supports the previous study that had suggested an increment in the club head speed score with the implementation of the active dynamic warm-up, warm-up with post-activation potentiation (PAP) technique, and the pre-shot routine [4, 5, 11, 13, 30] and increasing the athletes performance [6–8, 31].

Another factor related to the driving performance is the driving distance which varies according to the club head speed score [29]. This study found that the driving distance had little improvement according to the club head speed score after participating for six weeks in the programs. In addition, the findings of this study are similar to the previous research which found that the driving distance before practicing driving skills, pre-shot routine, and practicing driving skills in conjunction with pre-shot routine programs were not significantly different after the five-week training [13]. Based on the previous research, the amateur golf player's ability to play golf would not increase or change immediately after a 14-week physical and psychological skill training, but after 16 weeks [2]. It would appear that the duration of the training of the three programs in this study should be increased in order to allow the amateur golfers for a more adaptation. Although there was no statistical significant difference in the performances of the golfers after the sixth weeks, however, this study found that the driving distance was increased in all experimental groups especially the AF and PR programs with a rather distinct improvement (22.39 yards and 10.79 yards, respectively). An explanation for the improvement in the driving distance seen in this study by adding of AF program is that the effect of the warm-up training program throughout the sixth weeks led to the improved physiological factors such as strength, balance, and flexibility [4, 10, 28] and neurological activity of the skeletal muscles [11]. This, in turn, may have resulted in the greater coordination, the force production, and the driving performance in relation to the driving distance. This finding is similar to the previous study which found that the distance in the golf driving increased after the warm-up program [11]. An explanation for the improvement in the driving distance seen in this study by adding of the PR program is that focusing on various activities associated with the driving led to the improved concentration during the driving. Besides, the practice of pre-shot routine also allowed the movement mechanism while hitting the ball to work more automatically [32]. As a result, swinging the ball to the sweet spot was more accurate and the smash factor was increased. These two factors affected the increase in the driving distance [11]. As mentioned above, it can be seen that the effect of AF and PR program trainings is consistent with the results of the past studies. However, when the training program which combined these two types of the exercises, a little improvement in the driving distance (4.1 yards) was found. There are a number of factors that could have contributed to this outcome. The complexity of the training program, the number of the activities, and the duration of the training could cause the amateur golfers not to connect the skills and led to the obvious changes. This supports the previous research which suggested that the amateurs may immediately recognize and learn new strategies and tactics that excellent athletes used but obvious developments and changes would occur after more than 14 weeks of training [33].

The last factor of this study related to the driving performance is the driving accuracy. Results showed no statistically significant differences between the three programs. Thus, the cumulative effects of training the three programs for six weeks on the driving accuracy cannot be supported. An existing study reported that the advantageous effect of the 14-week cognitive-behavioral intervention program did not immediately improve the performance in the elite collegiate golfers. Improvements in the performance were reported in this particular study; however, in a 4-month follow-up, the researchers acknowledged that intervening variables may have confounded these improvements [2]. It has been suggested that the extended periods may be required for the internalization of cognitive-behavioral performance strategies [2, 33]. This may explain the findings in the present study, in that extended time may be required to relegate well-established strategies, and learn and adjust to new ones [2]. The previous study reported the significant improvements in putting performance among the novice golfers, utilizing a cognitive-behavioral intervention in the later stages of a 14-week study [33]. These improvements were maintained over a period, with a change in the behavior indicative of motor skill learning [13]. Although there was no statistically significant difference after the sixth weeks in which PR program according to previous research [20, 21, 23], however, this study found that the driving accuracy was increased in all experimental groups especially in the AF program, which had a rather distinct improvement (22.60 yards). An explanation for the improvement in the driving accuracy seen in this study by adding of AF program is that the effect of training the warm-up program throughout the six weeks led to improved physiological factors such as strength, balance, and flexibility [4, 28]. These are factors that create the power to hit the ball with distance and accuracy [34]. A disparate result for the cumulative effects of AFPR program may be the complexity of the training program and the amount of activity that was abundant. Thus, the training duration was not enough to connect the skills and change. Reasons for the ineffectiveness of the PR program may be the duration of the posttest which was determined one week after the training program to assess the accuracy that occurred after the training program had stopped. This study is different from the previous studies, which typically studied the effects of the pre-shot routine program in an acute phase by testing immediately after the program ended [13, 23]. Thus, the results of driving accuracy in this study are different from the previous studies.

Our results are in line with the later evidence showing that the limited time and the complex intervention dose inhibited a cumulative benefit. This implies that providing an amateur golfer with a single, easy-to-understand technique with limited time depends on their learning is a good option in the applied settings for intervention development because it is likely to give the amateur golfer to relegate the well-established strategies, and learn and adjust to the new ones. As noted above, the results suggested that the AFPR, PR, and AF developed as part of this study helped to improve the club head speeds, the driving distance, and the driving accuracy. Although the results of this study showed small increases in the club head speed, the driving distance, and the driving accuracy of the amateur golf players, however, the improvement was in a good direction and also supported the previous studies. The results of this study can be incorporated into the daily practice of the coaches with the amateur golfers when helping them to devise their driving training program. More research is required to identify the latent effect of these programs, the optimum duration of the warm-up and pre-shot routine, and the optimum components of the exercises such as the repetitions, the level of resistance, the equipment used, and the mental techniques used. Additionally, further research involving the use of these types of exercises in structured training programs over a longer period and their effect on the performance would also be beneficial.

## Conclusions

The findings of the present study showed that amateur golfers were able to improve the speed of the club head, the driving distance and the driving accuracy following a 6-week acquisition phase utilizing either AFPR, PR or AF program. However, statistically significant improvements in performance were not found in the 6-week programs. Although this study attempted to provide a robust design, there are limitations that may have affected our results such as the small number of the participants within the experimental groups, the complexity of the intervention, and the limited time of the training. Increasing the duration of the training is an option to improve the club head speed, the driving distance, and the driving accuracy. Moreover, the introduction of the program that combines pre-shot routine and warm-up training should be done after the amateur golfers have solely learned a pre-shot routine and warm-up programs, as the golfers have known how to use both techniques and have the skills to use them. Thus, the connection between the two techniques is more facile.

Further research may be carried out by increasing the duration of the training for more than 6 weeks to see the cumulative effects over the longer periods. The sample size should be increased to infer to the population more reliable. Besides, further study may be extended to the professional golfers to explore the effects on the different levels of the performance.
